# Metabolism-Associated DNA Methylation Signature Stratifies Lower-Grade Glioma Patients and Predicts Response to Immunotherapy

**DOI:** 10.3389/fcell.2022.902298

**Published:** 2022-06-15

**Authors:** Guozheng Yang, Dezhi Shan, Rongrong Zhao, Gang Li

**Affiliations:** ^1^ Department of Neurosurgery, Qilu Hospital, Cheeloo College of Medicine and Institute of Brain and Brain-Inspired Science, Shandong University, Jinan, China; ^2^ Department of Neurosurgery, Beijing Hospital, Chinese Academy of Medical Sciences, Graduate School of Peking Union Medical College, Beijing, China; ^3^ Shandong Key Laboratory of Brain Function Remodeling, Jinan, China

**Keywords:** metabolism, DNA methylation, CpG, lower-grade glioma, risk model, immune checkpoint inhibitor treatment

## Abstract

Metabolism and DNA methylation (DNAm) are closely linked. The value of the metabolism-DNAm interplay in stratifying glioma patients has not been explored. In the present study, we aimed to stratify lower-grade glioma (LGG) patients based on the DNAm associated with metabolic reprogramming. Four data sets of LGGs from three databases (TCGA/CGGA/GEO) were used in this study. By screening the Kendall’s correlation of DNAm with 87 metabolic processes from KEGG, we identified 391 CpGs with a strong correlation with metabolism. Based on these metabolism-associated CpGs, we performed consensus clustering and identified three distinct subgroups of LGGs. These three subgroups were characterized by distinct molecular features and clinical outcomes. We also constructed a subgroup-related, quantifiable CpG signature with strong prognostic power to stratify LGGs. It also serves as a potential biomarker to predict the response to immunotherapy. Overall, our findings provide new perspectives for the stratification of LGGs and for understanding the mechanisms driving malignancy.

## 1 Introduction

Lower-grade gliomas (LGGs) are common primary intracranial tumours characterized by inevitable recurrence, high mortality and substantial heterogeneity (van den Bent, 2010). Due to the substantial heterogeneity of the tumour, different individuals usually have highly variable prognoses and distinct responses to various types of treatments. Although the World Health Organization (WHO) classification has incorporated molecular markers, such as isocitrate dehydrogenase (IDH) mutation, chromosome 1p/19q codeletion, and TERT promoter mutation, which has greatly improved the stratification of glioma patients (Louis et al., 2016), the heterogeneity of gliomas still needs to be further elucidated.

Metabolic reprogramming is a hallmark of cancer (Faubert et al., 2020). It has been proven to affect cancer cell proliferation, migration and invasion in multiple ways, such as satisfying the increased energy needs of cancer cells and affecting various biological processes by altering the level of substrates. Epigenetic modification is a process that regulates gene expression without altering DNA sequences. It is plastic, inheritable and plays a pivotal role in cell differentiation and cell function (Bauer et al., 2016; Gu L. et al., 2020; Blanco et al., 2021). Recent studies have also found that it plays important roles in disease pathogenesis and diagnosis (Taby and Issa, 2010; Gu et al., 2015). As an important form of epigenetic modification, DNA methylation (DNAm) has been proven to be involved in carcinogenesis (Hao et al., 2017; Hu et al., 2021). It is a process in which a methyl group is transferred onto the C5 position of the cytosine to form 5-methylcytosine (5-mC) (Schübeler, 2015). The methylation process is mediated by DNA methyltransferases (DNMTs), while the demethylation process is driven by ten-eleven translocation (TET) enzymes. In addition to DNMTs and TET enzymes, methylation can also be regulated by metabolism, and they are closely interlinked. Recent advances have shed light on the reciprocal regulatory relationship between metabolism and DNAm in cancer cells (Lopes, 2020; Sun et al., 2021). On the one hand, metabolic changes can affect DNAm by altering the levels of substrates. For example, in IDH-mutant tumours, the aberrant metabolite 2-hydroxyglutarate (2-HG), produced in the tricarboxylic acid cycle, can inhibit TET-mediated DNA demethylation, thus causing genome hypermethylation (Xu et al., 2011). S-adenosyl methionine (SAM), produced in one-carbon metabolism processes such as the methionine cycle and the folate cycle, is the major methyl group donor of DNAm, and a low-folate diet has been reported to cause DNA hypomethylation and increase the risk of neoplasia (Crider et al., 2012). On the other hand, DNAm regulates the expression of metabolism-associated genes by affecting chromatin structure and transcription factor binding (Pan et al., 2018). In recent years, the development of computer science and omics technology has brought new opportunities to cancer research, allowing us to use bioinformatic methods, such as machine learning, to conduct analyses on high-throughput omics data and perceive disease molecular differences more comprehensively (Wang et al., 2013; Gu H. et al., 2020). The aforementioned studies on metabolism and methylation mainly focused on the regulatory mechanisms of specific metabolites on DNAm, and much effort has been devoted to exploring the metabolic heterogeneity of tumours from the perspective of transcriptomics and metabolomics (He et al., 2020; Gong et al., 2021). No one has studied the value of the metabolism-DNAm interplay in cancer stratification.

In the present study, we aimed to study the value of metabolism-associated DNAm in the stratification of LGGs. We conducted an integrated analysis of methylome and transcriptome data from The Cancer Genome Atlas (TCGA), Gene Expression Omnibus (GEO) and Chinese Glioma Genome Atlas (CGGA) databases and an independent cohort (Qilu cohort). The correlation between DNAm and metabolism was evaluated. Three biologically discrete subgroups with distinct metabolism-associated DNAm patterns were identified. In addition, we constructed a subgroup-related, quantifiable CpG signature to stratify patient prognosis and predict the potential response to immune checkpoint inhibitor (ICI) treatment, providing a novel biomarker for precision medicine. Together, our analyses highlighted the previously unappreciated role of DNAm in metabolism-associated heterogeneity in LGGs.

## 2 Materials and Methods

### 2.2 Data Collection and Processing

#### 2.1.1 Publicly Available Datasets

The methylation, gene expression and clinical data of the TCGA LGG cohort were collected from the UCSC Xena database, and the methylation and clinical data of the GSE48461 and GSE104293 data sets were collected from the GEO database. The mRNAseq_693 data set of the CGGA database was also collected. Only patients with primary LGGs (WHO grade II and grade III) in the above data sets were included for analysis (baseline characteristics are summarized in Supplementary Table S1). Methylation data were filtered and normalized using the champ.filter and champ.norm functions of the ‘ChAMP’ R package. For quality control, outliers shown in principal component analysis (PCA) were excluded. Furthermore, CpGs that were hypermethylated (β > 0.7) or hypomethylated (β < 0.3) in 98% of all samples were also excluded. Only CpGs that existed in both the TCGA and GEO data sets were included. Batch effects of methylation data among the TCGA LGG, GSE48461, and GSE104293 data sets were removed using the “sva” R package according to a previously reported pipeline (Marabita et al., 2013). GSE48461 and GSE104293 were merged into one cohort and considered the GEO cohort. For transcriptome data, frglycoagments per kilobase of transcript per million mapped reads (FPKM) format data were transformed into transcripts per million (TPM) format for analysis.

#### 2.1.2 Patient Sample Collection of the Qilu Cohort

We collected samples for mRNA sequencing from 23 LGG patients who received surgical resection at Qilu Hospital of Shandong University from May 2018 to December 2018. Transcriptome data were transformed into TPM format for analysis. The study was approved by the Research Ethics Committee of Qilu Hospital of Shandong University, and written informed consent was obtained from all patients.

### 2.2 Bioinformatic Analysis

Gene set variation analysis (GSVA) is a method for estimating the variation in gene set enrichment through the samples of a transcriptome data set. Various biological process-associated gene sets were collected from the Molecular Signatures Database (MSigDB) and previous studies (Rosario et al., 2018). The GSVA score of each biological process for each sample was calculated using the ‘GSVA’ R package.

Gene Ontology (GO) enrichment analysis is a method to annotate gene sets. It finds the overrepresented GO terms of a given set of genes based on the annotations of the gene set. Gene set enrichment analysis (GSEA) is a computational method to determine whether a given set of genes has significant differences in two groups of objects. Both GO enrichment analysis and GSEA were conducted using the “clusterProfiler” R package.

Differentially expressed genes (DEGs) between groups were identified using the “limma” R package on count data, and genes with absolute log2-fold change (|log2FC|) > 2 and *adjP* <0.05 were defined as significantly differentially expressed. Differentially methylated CpGs (DMCs) were determined using the champ.DMP function of the “ChAMP” package.

### 2.3 Metabolism-Associated CpGs in Lower-Grade Gliomas

First, 87 metabolic processes acquired from the Kyoto Encyclopedia of Genes and Genomes (KEGG) database were quantified using the GSVA algorithm. Kendall’s correlation was used to assess the correlation between metabolic processes and CpGs. IDH mutation has a profound effect on both metabolism and DNAm (Han et al., 2020). Such an effect could be a significant confounding factor in assessing the correlation between metabolism and DNAm. Thus, in this part, we included only IDH wild-type LGGs from the TCGA (*n* = 91) for metabolism-associated CpG identification. Kendall’s tau-b (τb) of each metabolic process with all CpGs was calculated based on GSVA scores and beta values. Univariate Cox regression was used to assess the association of CpGs with overall survival (OS). Then, the criteria below were used to eventually determine the metabolism-associated CpGs: 1) Kendall’s tau-b (τb) > 0.4 and false discovery rate (*FDR*) < 0.05; 2) prognostic significance in both IDH-mutant and wild-type LGGs (univariate Cox *p value* < 0.05).

### 2.4 Clustering, Classification of Lower-Grade Gliomas and Validation

To identify metabolism-associated DNAm subgroups, K-means clustering was carried out based on the metabolism-associated CpGs using the R package “ConsensusClusterPlus”. The similarity between samples was measured by Euclidean distance. Eighty percent of the samples were resampled 1000 times. The optimal k value was determined by the cumulative distribution function (CDF).

For external validation of the clinical characteristics of the clustering, we used the K-nearest neighbour (KNN) algorithm to classify the GEO cohort. The KNN algorithm is a commonly used machine learning classification algorithm. It takes k training samples closest to the object as a reference and assigns the object to the most common class among them. In the present study, to determine the optimal k value, the TCGA cohort was randomly assigned to a training set and testing set at a ratio of 1:1. The classification accuracy of KNN classification when k ranged from 1 to 20 was tested, and k value corresponding to the highest accuracy was determined as the optimal k value. Then, taking the clustered TCGA cohort as a reference, the GEO cohort was classified by the KNN method, and clinical characteristics were assessed. PCA was also performed to validate the clustering and classification results.

### 2.5 Construction of the Metabolic CpG Signature

For the construction of the metabolic CpG signature, differential methylation analysis was performed on the TCGA cohort using the DMP function of the “ChAMP” R package. Differences were considered significant if delta-beta > 0.1 and *FDR* < 0.05. Metabolic-related CpGs differentially methylated across all subgroups were selected and considered subgroup-related CpGs. Then, least absolute shrinkage and selection operator (LASSO) Cox regression was performed on these CpGs. The optimal lambda value was determined according to the partial likelihood deviance. The risk score of each sample was defined as the sum of the beta values of the signature CpGs weighted by their LASSO coefficients. The risk score can be calculated using the following equation, where *Beta* and *Coef* represent the beta value of the CpG and the corresponding coefficient, respectively:

$$RiskScore=\overset{M}{\underset{i=1}{\sum }}\left(Beta_{i}*Coef_{i}\right)$$

An alternative gene signature was also generated by fitting gene expression to the CpG signature-generated risk score. Specifically, Kendall’s correlation of all genes with the risk score was caclulated. Genes with a strong linear correlation (τb > 0.35, *FDR* < 0.05) with the risk score were selected. LASSO regression was utilized to fit these genes to the risk score. The optimal lambda value was determined according to the mean-squared error. The risk score can be calculated using the following equation, where *GeneExp* represents gene expression, and *Coef* represents the corresponding coefficient:

$$RiskScore=\overset{M}{\underset{i=1}{\sum }}\left(GeneExp_{i}*Coef_{i}\right)+Intercept$$

### 2.6 Correlation Between the Signature and Ki-67 Positive Rate

Immunohistochemistry-determined Ki-67 positive rate is a widely used indicator of the percentage of proliferating cells within a tumour. To further validate our signature, we compared the Ki-67 positive rate determined by immunohistochemistry and the risk score using the Qilu cohort. Specifically, the Ki-67 positive rates were determined by two experienced pathologists from the Department of Pathology of Qilu Hospital and were then divided into lower positive rates (<10%) and higher positive rates (≥10%). The alternative gene signature was applied to calculate the risk scores of the Qilu cohort. The Qilu cohort was divided into a high-risk group and a low-risk group using the median risk score as the cut-off value. Finally, Fisher’s exact test was used to determine the significance of the difference in the Ki-67 positive rate between different groups.

### 2.7 Estimation of Immune Infiltration

ESTIMATE was used to quantify the overall immune and stromal abundance of samples. Single-sample gene set enrichment analysis (ssGSEA) and MCP-counter were used to quantify immune subpopulations. Immune metagenes for ssGSEA were collected from a previously published study (Charoentong et al., 2017).

### 2.8 Prediction of Immunotherapy Response

The Immunophenoscore (IPS) is an algorithm that predicts the response to ICI treatments. It quantifies the potential immunotherapy response through four aspects, including MHC-related molecules (MHC), checkpoints or immunomodulators (CP), effector cells (EC) and suppressor cells (SC), and integrates them into the IPS. A higher IPS indicates a better response to ICI therapy, while a lower IPS indicates a poorer response. An online tool to calculate the IPS was developed by the authors (https://tcia.at/).

### 2.9 Statistical Analysis

The Wilcoxon test and Student’s t test were used to assess differences in continuous variables among/between groups. Fisher’s exact test and the chi-square test were used to assess differences in contingency table variables among/between groups. Multivariate Cox regression was implemented to determine the independence of prognostic factors. The Kaplan-Meier method and the log-rank test were used to assess the significance of prognostic differences among/between groups. Kendall’s tau-b was used to assess the correlation between variables. Statistical significance was defined as *p* < 0.05. All analyses were performed using R software version 4.1.1 (https://www.r-project.org/).

## 3 Results

### 3.1 Identification of Metabolism-Associated DNAm Patterns in Lower-Grade Gliomas

**FIGURE 1:**
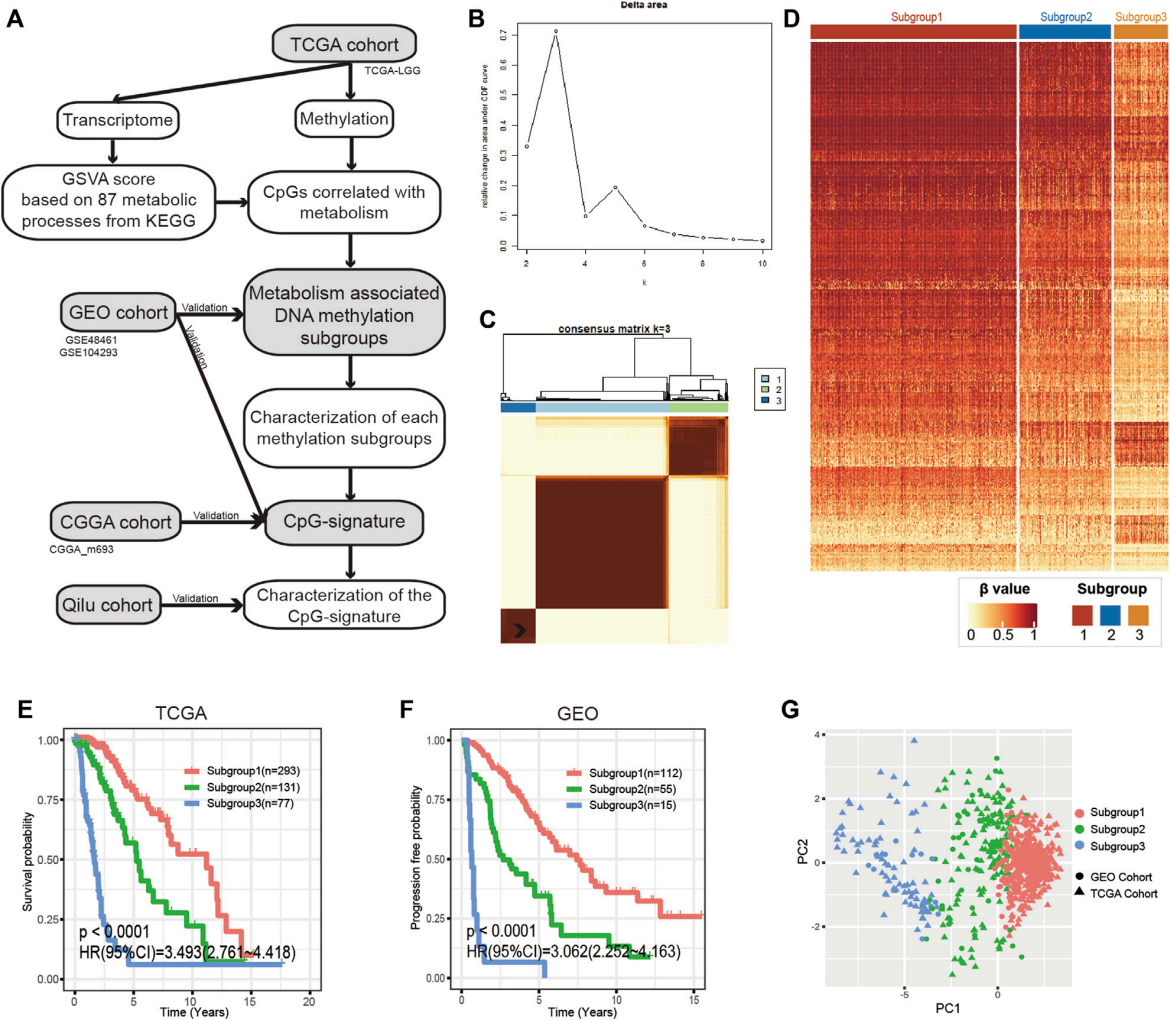
Identification of metabolism-associated DNA methylation patterns. **(A)** Flowchart of the research. **(B)** Relative changes in the area under the CDF curve when k ranges from 2 to 10. **(C)** Consensus matrix heatmap of the three distinct subgroups in the TCGA cohort. **(D)** Heatmap of metabolism-associated CpGs in the three subgroups of the TCGA cohort. **(E,F)** Kaplan-Meier analysis of the three subgroups in the TCGA cohort and the GEO cohort. **(G)** PCA of metabolism-associated CpGs to demonstrate the three subgroups in the TCGA and GEO cohorts. CDF, cumulative distribution function; PCA, principal component analysis.

We created a flowchart to demonstrate the workflow of our research (Figure 1A). According to Kendall’s correlation coefficient and the selection criteria described in the Methods and Materials, we acquired 708 metabolic process-CpG pairs consisting of 35 metabolic pathways and 391 CpGs (Supplementary Figures S1A,B and Supplementary Table S2). Based on the 391 CpGs, we performed k-means clustering on the TCGA cohort. According to the CDF, we chose k = 3 as the optimal number of clusters (Figure 1B and Supplementary Figure S1C). As shown in the heatmap (Figures 1C,D), 501 LGG patients were clustered into three distinct subgroups. We then applied Kaplan-Meier analysis to investigate the prognostic significance of the clustering. The results showed significant differences in OS among the three subgroups (Figure 1E). To further validate the prognostic value of such methylation patterns, we assigned the GEO cohort (GSE48461 and GSE104293) to the aforementioned subgroups using the KNN algorithm. To determine the optimal k value of KNN, the TCGA cohort was first randomly assigned to the training set and testing set at a ratio of 1:1, and the accuracy of KNN classification when k ranged from 1 to 20 was tested. According to the test, KNN had the best classification accuracy (94%) when k = 3 (Supplementary Figure S1D). Then, taking the TCGA cohort as a reference, we assigned the GEO cohort to the three distinct subgroups (Supplementary Figure S1E). Subsequent Kaplan-Meier analysis also showed significant differences in progression-free survival (PFS) among the subgroups (Figure 1F). Moreover, PCA was performed to further validate the subgrouping of both the TCGA and GEO cohorts (Figure 1G).

### 3.2 The Distinct Lower-Grade Glioma Subgroups Present Different Biological Characteristics

**FIGURE 2:**
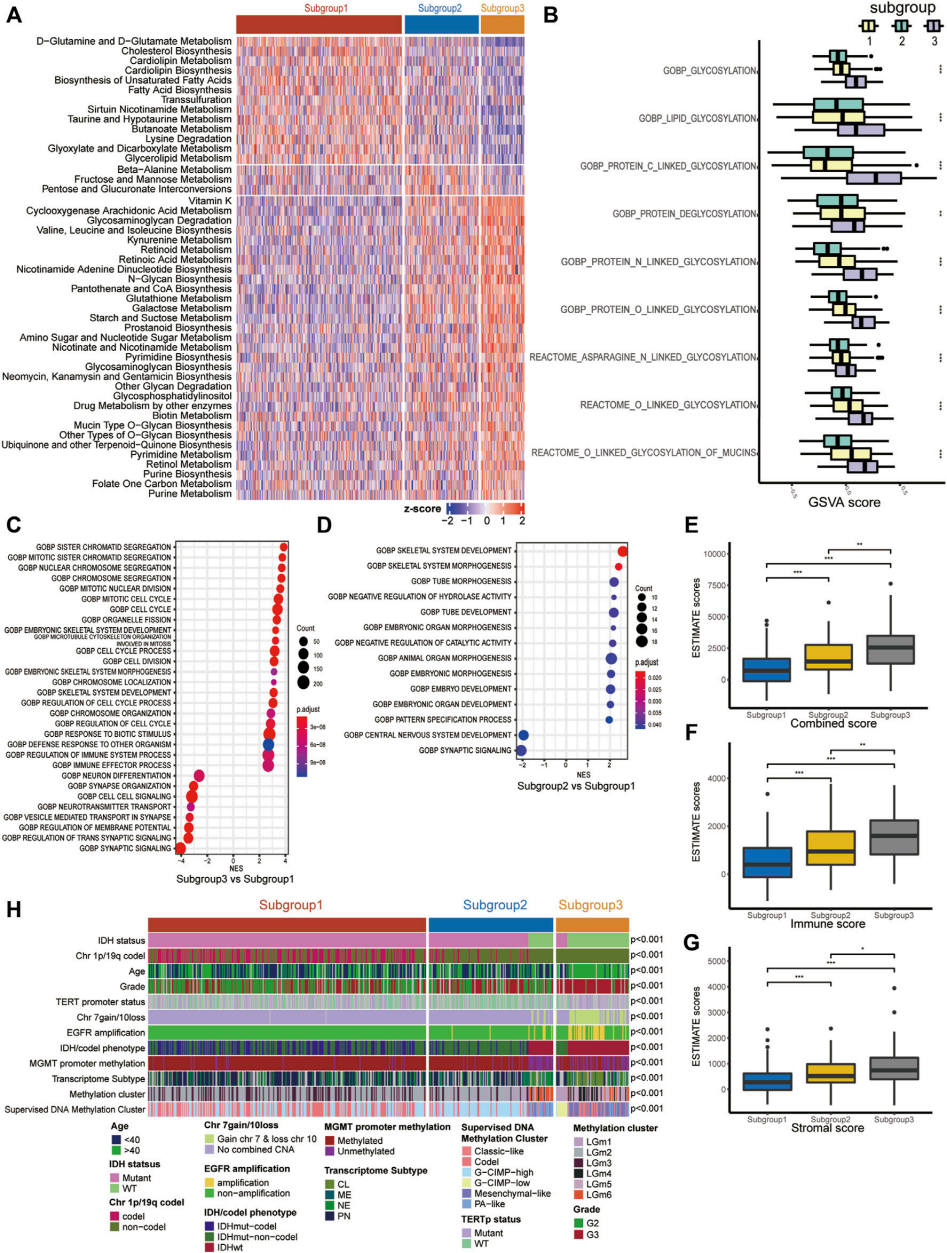
Molecular characteristics of the three LGG subgroups. **(A)** Heatmap of the subgroup-specific metabolic signatures. **(B)** Boxplot of the GSVA scores of glycosylation processes. **(C,D)** Dotplot of functional analysis using GSEA. **(E–G)** Boxplot of the combined score, immune score and stromal score estimated using ESTIMATE. **(H)** Correlation of our subgroups with common clinical features/molecular subclasses. ns represents no significance, *p < 0.05, **p < 0.01, ***p < 0.001.

Since our classification is based on metabolism, we explored the metabolic features of each subgroup. First, 114 metabolic signatures were acquired from a previously published study. We used GSVA to quantify the 114 metabolic signatures. Then, we performed differential analysis to determine subgroup-specific metabolic signatures, which were defined as signatures with the highest GSVA scores in certain subgroups. Eventually, 31 subgroup-specific metabolic signatures were identified (Figure 2A). Subgroup 1, subgroup 2 and subgroup 3 had 13, 3 and 31 subgroup-specific metabolic signatures, respectively. Subgroup 1 had higher scores in the glutamine and glutamate metabolism and lipid metabolism signatures. Notably, 8 of 31 subgroup-specific signatures of subgroup 3 were associated with glycan metabolism, such as glycosaminoglycan degradation and mucin-type o-glycan biosynthesis. Based on this result, we further investigated whether subgroup 3 had a more active glycosylation process, which has been proven to promote malignancy in various types of neoplastic diseases. As expected, we found that the GSVA scores of glycosylation processes in subgroup 3 were significantly higher than those in the other subgroups (Figure 2B).

To further clarify the underlying biological characteristics of each subgroup, we performed GSEA among the subgroups. Specifically, we performed differential expression analysis among all three subgroups. DEGs were defined as |log2FC| > 1 and *adjP* < 0.05. Then, we ranked the DEGs according to the log2FC and performed GSEA. The results suggested that subgroup 3, with the worst prognosis, was significantly enriched in cell cycle- and immune-related processes (Figure 2C, Supplementary Figure S2A). Subgroup 2, with a moderate prognosis, was mainly enriched in embryonic development-related processes relative to subgroup 1 (Figure 2D).

### 3.3 Correlation of the Lower-Grade Glioma Subgroups With Clinical and Molecular Features

We also investigated the relationship between our subgroups and common clinical features/molecular subclasses of LGGs, including histological grade, IDH mutation, MGMT promoter methylation, TERT promoter mutation, and combined chromosome 7 +/10− events (Figure 2H and Supplementary Table S3). In the TCGA cohort, we found that subgroup 1 consisted of only IDH-mutant LGGs and was closely associated with 1p/19q codel subset (*p* < 0.001), PN transcriptome phenotype (*p* < 0.001), TERT promoter mutation (*p* < 0.001), etc. In IDH-mutant LGGs of the TCGA cohort, subgroup 2 was significantly linked with chromosome 1p/19q non-codeletion (*p* < 0.001) and the G-CIMP-high subset. Subgroup 3 was closely associated with histological grade III (*p* < 0.001), methylated MGMT promoter (*p* = 0.0308), chromosome 1p/19q non-codeletion (*p* < 0.001) and the G-CIMP-low phenotype of the TCGA supervised DNA methylation cluster. In IDH wild-type LGGs of the TCGA cohort, subgroup 2 was correlated with histological grade II, PA-like subset of the TCGA supervised DNA methylation cluster (*p* < 0.001) and NE transcriptome subtype (*p* < 0.001), while subgroup 3 was significantly correlated with chromosome 7 gain/10 loss, TERT promoter mutation, EGFR amplification, and classic-like and mesenchymal-like subsets of the TCGA supervised DNA methylation cluster (*p* < 0.001).

To explore whether the distinct subgroups were accompanied by different immune infiltration levels, we quantified the immune infiltration abundance of each subgroup using the ESTIMATE algorithm. As a result, significant differences in immune scores, stromal scores and combined scores were observed among the subgroups, with the highest scores in subgroup 3, followed by subgroup 2, and the lowest in subgroup 1 (Figures 2E–G).

### 3.4 Construction of the Metabolic CpG Signature

**FIGURE 3:**
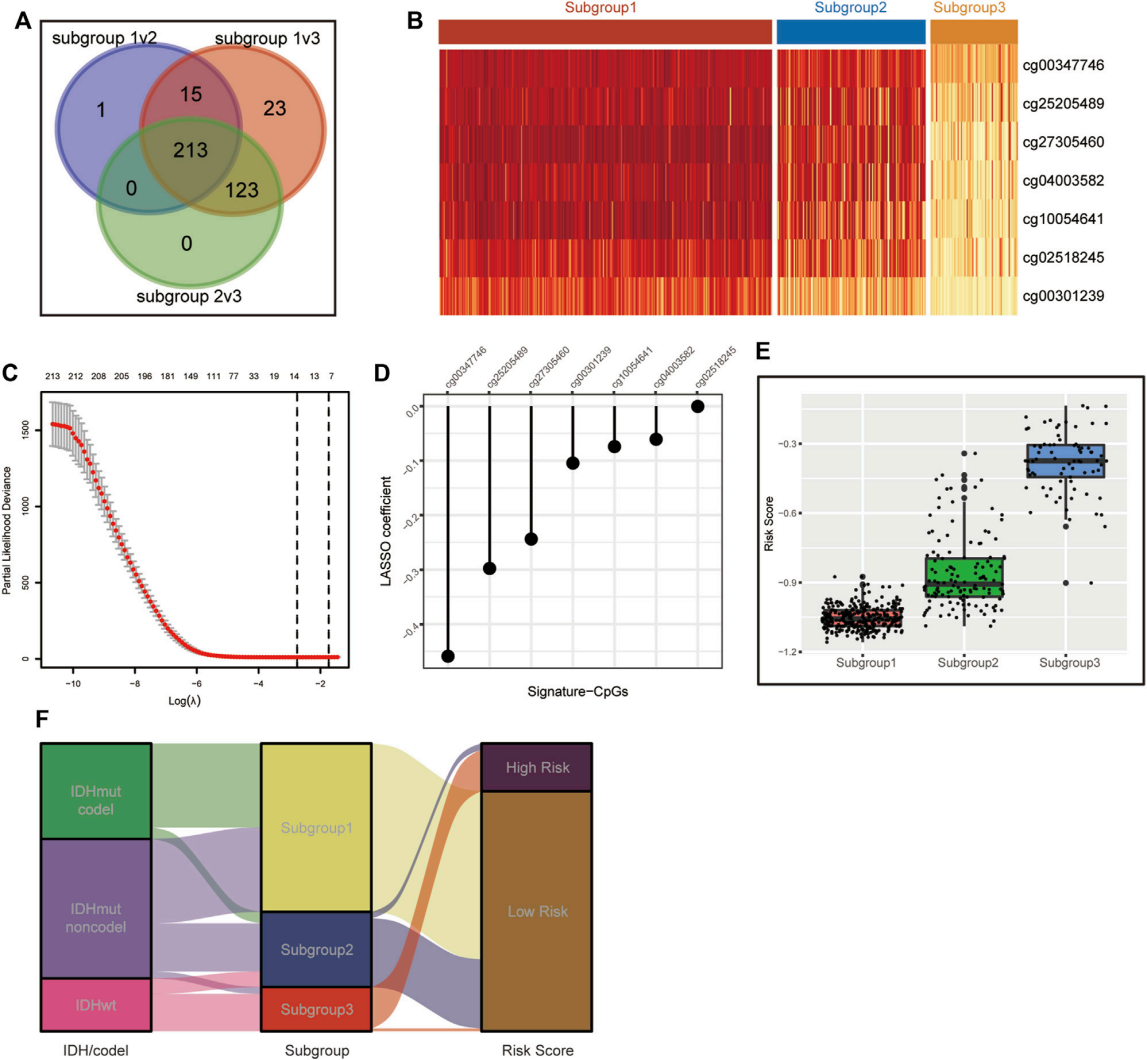
Construction of the CpG signature. **(A)** Venn diagram illustrating the 213 metabolism-associated CpGs differentially methylated across all three subgroups. **(B)** Heatmap of beta values of the signature CpGs in the three LGG subgroups in TCGA. **(C)** Plot of correlation between partial likelihood deviance and lambda value in LASSO regression. The maximum lambda value within the one standard error of the partial likelihood deviance minimizer was determined as the optimal value. **(D)** Signature CpGs and corresponding LASSO coefficients. **(E,F)** Boxplot **(E)** and Sankey diagram **(F)** demonstrating the correlation of the CpG signature and metabolism-associated subgroups in TCGA.

To simplify our subgrouping for practical application, we constructed a subgroup-associated scoring scheme termed the metabolic CpG signature. For the construction of the signature, we first performed differential methylation analysis among the subgroups on metabolism-associated CpGs (differences were considered significant if *FDR* < 0.05 and Δβ > 0.1). CpGs differentially methylated in all possible comparisons were considered subgroup-related CpGs. Eventually, 213 subgroup-associated CpGs were identified (Figures 3A,B). Next, we performed LASSO regression on these subgroup-associated CpGs. After comprehensive consideration of the parsimony and generality of the signature, we chose the maximum lambda value within one standard error of the value that minimizes the partial likelihood deviance as the optimal value (Figure 3C). As a result, a CpG signature consisting of seven CpGs was generated (Figure 3D and Supplementary Table S4).

**FIGURE 4:**
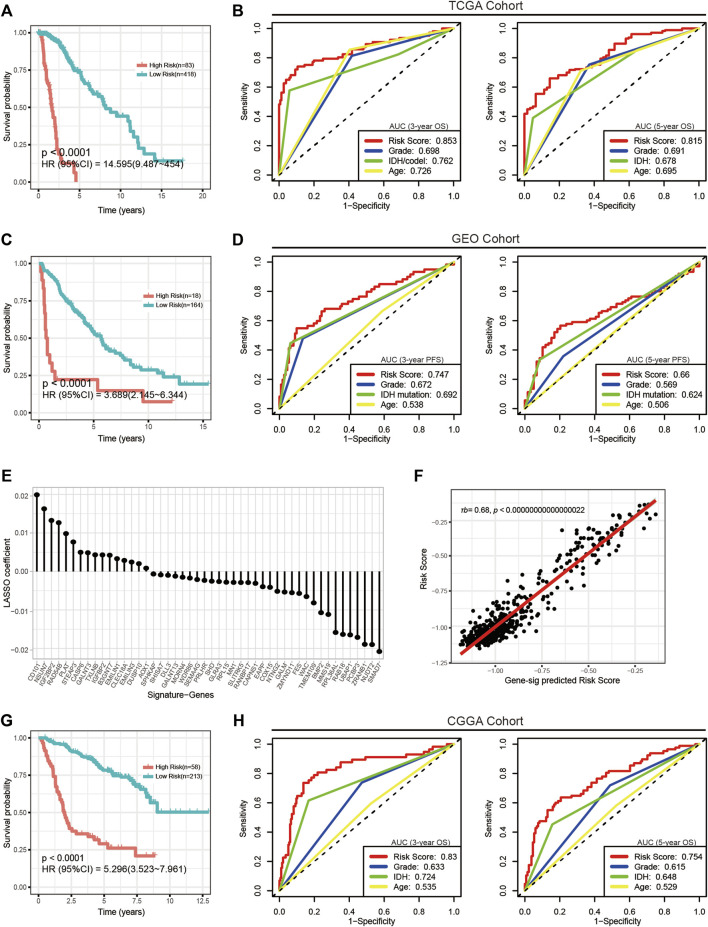
Evaluation of the CpG signature in the TCGA, GEO and CGGA cohorts. **(A,G)** Kaplan-Meier analysis stratifying overall survival in the TCGA **(A)** and CGGA **(G)** cohorts. **(C)** Kaplan-Meier analysis stratifying progression-free survival in the GEO cohort. **(B,H)** ROC curves and AUC values showing the performance in predicting 3-year (left) and 5-year (right) survival in the TCGA and CGGA cohorts. **(D)** ROC curves and AUC values showing the performance in predicting 3-year (left) and 5-year (right) progression. **(E)** Signature genes and corresponding coefficients in the alternative gene signature. **(F)** Plot of the correlation between the alternative gene signature predicted risk scores and actual risk scores calculated by the CpG signature. τb, Kendall’s tau-b.

We calculated the risk scores of all TCGA samples. Analyses showed that the risk score was significantly differentially distributed among the three subgroups. Most subgroup 3 LGGs were assigned to the high-risk group, and most of subgroup 1 and subgroup 2 LGGs were assigned to the low-risk group. (Figures 3E,F). We then assessed the prognostic significance and efficacy of the signature. The TCGA cohort was first divided into high-risk and low-risk groups. We determined −0.604472 as the optimal cut-off value using the surv_cutpoint function of the “survminer” R package. Patients with risk scores higher than the cut-off were assigned to the high-risk group, while those with risk scores lower than the cut-off were assigned to the low-risk group. Kaplan-Meier analysis confirmed that the signature was capable of stratifying the prognoses of LGG patients (Figure 4A). Multivariate Cox regression considering age, IDH mutation, chromosome 1p/19q codeletion and histologic grade confirmed the independence of the signature (Table 1). A receiver operating characteristic (ROC) curve was generated to evaluate the prognostic power of our signature. The area under the curve (AUC) suggested that our signature had great prognostic power (0.853 in predicting 3-year OS, 0.815 in predicting 5-year OS) and performed better than common clinical features, such as IDH mutation, grade, and age (Figure 4B).

**TABLE 1:** Univariate and multivariate Cox regression analysis of risk score and other clinical features in the TCGA/CGGA/GEO cohorts.

Variables	Univariate analysis		Multivariate analysis	
	HR (95% CI)	*p-*value	HR (95% CI)	*p*-value
*TCGA cohort*				
Risk score	14.595 (9.487–22.454)	3.66E-30	10.829 (5.154–22.754)	**3.21E-10**
Age	3.119 (2.096–4.640)	5.19E-09	2.235 (1.431–3.490)	**4.04E-04**
IDH mutation	6.307 (4.325–9.198)	4.10E-18	0.657 (0.326–1.323)	2.39E-01
Grade	3.312 (2.227–4.926)	4.36E-10	2.183 (1.415–3.367)	**4.15E-04**
1p/19q codel	0.400 (0.250–0.641)	2.95E-05	0.615 (0.364–1.040)	6.97E-02
*GEO cohort*				
Risk score	3.689 (2.145–6.344)	4.37E-05	6.620 (1.102–39.775)	**3.88E-02**
Age	1.101 (0.743–1.632)	6.30E-01	1.028 (0.692–1.527)	8.90E-01
IDH1 mutation	0.254 (0.166–0.389)	1.49E-08	0.471 (0.227–0.976)	**4.28E-02**
Grade	1.961 (1.288–2.986)	2.57E-03	1.049 (0.627–1.754)	8.56E-01
*CGGA cohort*				
Risk Score	5.296 (3.523–7.961)	1.26E-13	3.641 (1.977–6.705)	**3.36E-05**
Age	1.154 (0.776–1.718)	4.78E-01	1.492 (0.947–2.350)	8.42E-02
IDH mutation	3.168 (2.082–4.820)	3.30E-07	0.831 (0.653–2.218)	5.53E-01
Grade	2.506 (1.625–3.863)	1.38E-05	3.125 (1.886–5.178)	**9.70E-06**
1p/19q codel	0.202 (0.107–0.381)	3.96E-09	0.321 (0.159–0.650)	**1.57E-03**

P-values marked in bold were considered significant statistically(*p* < 0.05).

### 3.5 External Validation of the CpG Signature

Next, we performed external validation in the GEO and CGGA cohorts. Of note, OS information was not provided in most publicly available LGG methylation data sets, and in the GEO cohort, only complete PFS information but incomplete OS information was available. To determine the signature’s performance in predicting PFS, we calculated risk scores for all patients in the GEO cohort and assigned them to the high-risk group and the low-risk group. Kaplan-Meier analysis and multivariate Cox regression confirmed the prognostic significance (Figure 4C) and independence of the signature (Table 1). Although ROC analysis showed that the metabolic CpG signature had good prognostic power in predicting 3-year PFS (AUC = 0.747) but not as good performance in predicting 5-year PFS (AUC = 0.66), the performance was better than that of common clinical features such as age, grade and IDH mutation (Figure 4D).

To validate the performance of the signature in predicting OS, we performed indirect external validation in the CGGA cohort by fitting gene expression to the risk score to construct a gene signature as an alternative. Specifically, we screened genes with a strong linear correlation with the risk score (τb > 0.35, FDR <0.05) and then fitted these genes to the risk score using LASSO regression (Supplementary Figures S4A,B). The maximum lambda value within one standard error of the mean-squared error minimizer was considered the optimal value. As a result, a gene signature consisting of 48 genes as an alternative to the CpG signature was generated (Figure 4E and Supplementary Table S4). The risk score calculated using this alternative gene signature had a strong correlation with that calculated using the CpG signature (Figure 4F). We then calculated risk scores for the CGGA cohort using the gene signature. Kaplan-Meier analysis (Figure 4G), multivariate Cox regression (Table 1), and ROC curves (Figure 4H) generated similar results to those from the TCGA data set; the AUCs were 0.83 for predicting 3-year OS and 0.754 for predicting 5-year OS. The results indicated that the signature was applicable to a wider range of cohorts.

### 3.6 Characterization of the Metabolic CpG Signature

**FIGURE 5:**
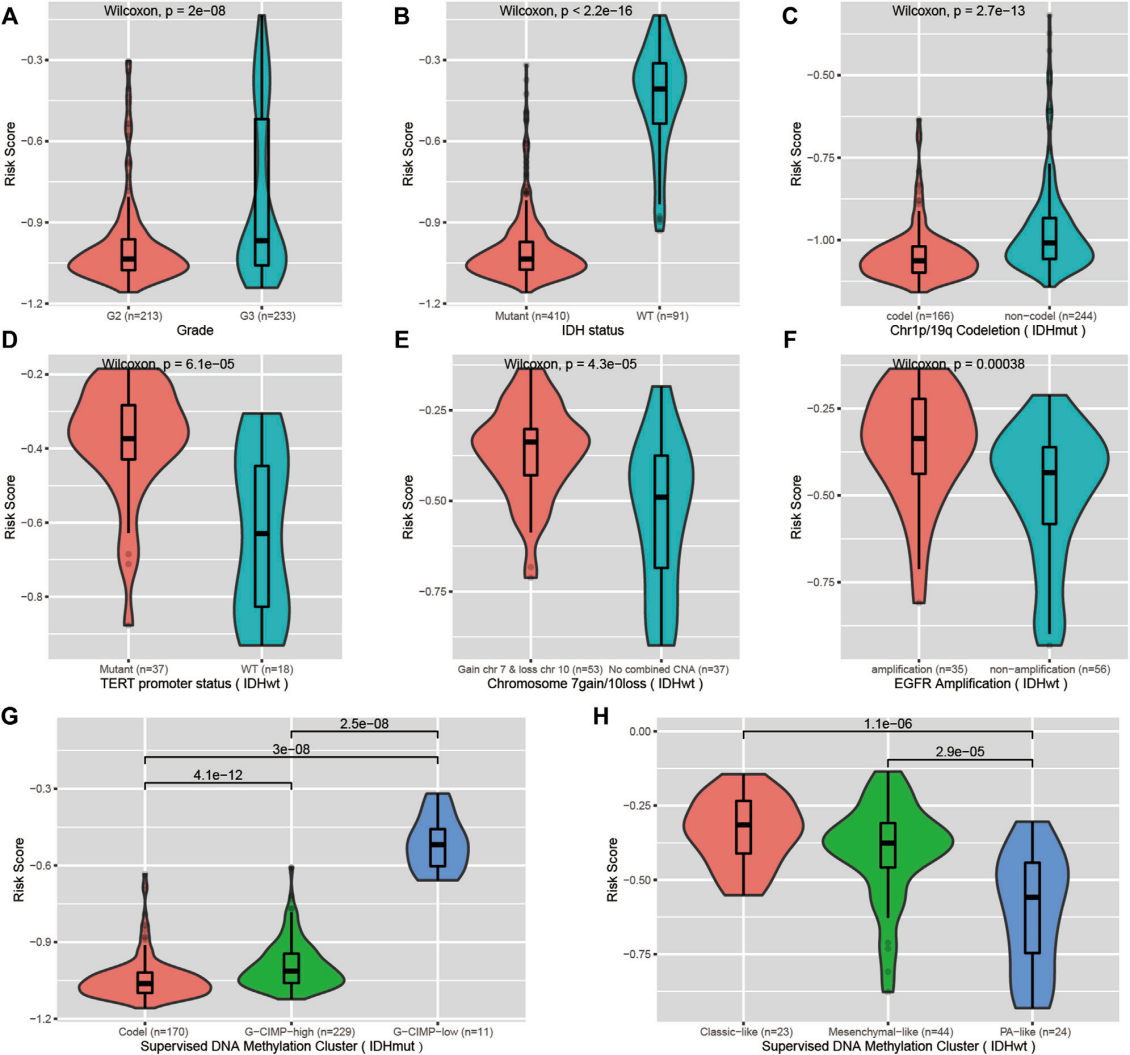
Correlation of the CpG signature with common clinical features. **(A,B)** Distribution of the risk scores in LGGs stratified by histological grade and IDH mutation status. **(C,G)** Distribution of the risk scores in IDH-mutant LGGs stratified by chromosome 1p/19q codeletion status and supervised DNA methylation cluster. **(D–F,H)** Distribution of the risk scores in IDH wild-type LGGs stratified by TERT promoter status, chromosome 7gain/10loss, EGFR amplification and supervised DNA methylation cluster.

**FIGURE 6:**
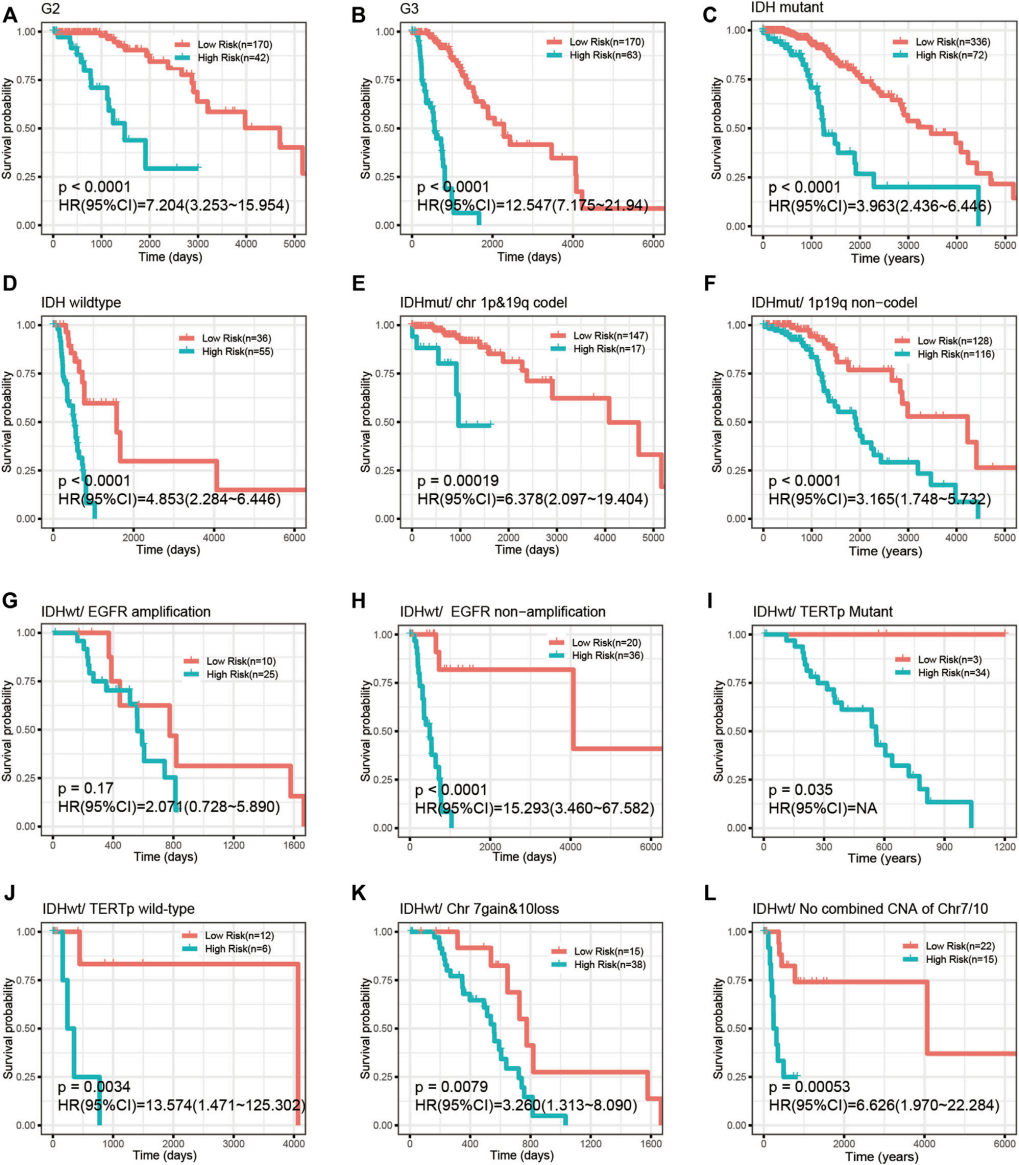
The CpG signature remained prognostically significant in LGGs stratified by common variables. **(A–D)** Kaplan-Meier analysis of the CpG signature in LGGs stratified by histological grade **(A,B)** and IDH mutation **(C,D)**. **(E,F)** Kaplan-Meier analysis of the CpG signature in chromosome 1p/19q codeletion-stratified IDH-mutant LGGs. **(G–L)** Kaplan-Meier analysis of the CpG signature in IDH wild-type LGGs stratified by EGFR amplification, TERT promoter mutation and combined chromosome 7gain/10loss.

We investigated the correlation of the risk score with common prognostic variables or molecular subclasses, including histological grade, IDH mutation, TCGA supervised DNA methylation cluster and chromosome 1p/19q codeletion status in IDH-mutant LGGs as well as chromosome 7loss/10gain, EGFR amplification, TERT promoter mutation in IDH wild-type LGGs. We noticed that the risk score had a significant difference in distribution between/among subgroups stratified by these common variables or subcategories. Grade III, IDH wild-type, chromosome 1p/19q non-codeletion, EGFR amplification, chromosome 7loss/10gain, and TERT promoter mutation all presented elevated risk scores relative to their respective counterpart subgroups (Figures 5A–H). Our signature was able to further stratify these stratified subgroups (Figure 6A–L).

**FIGURE 7:**
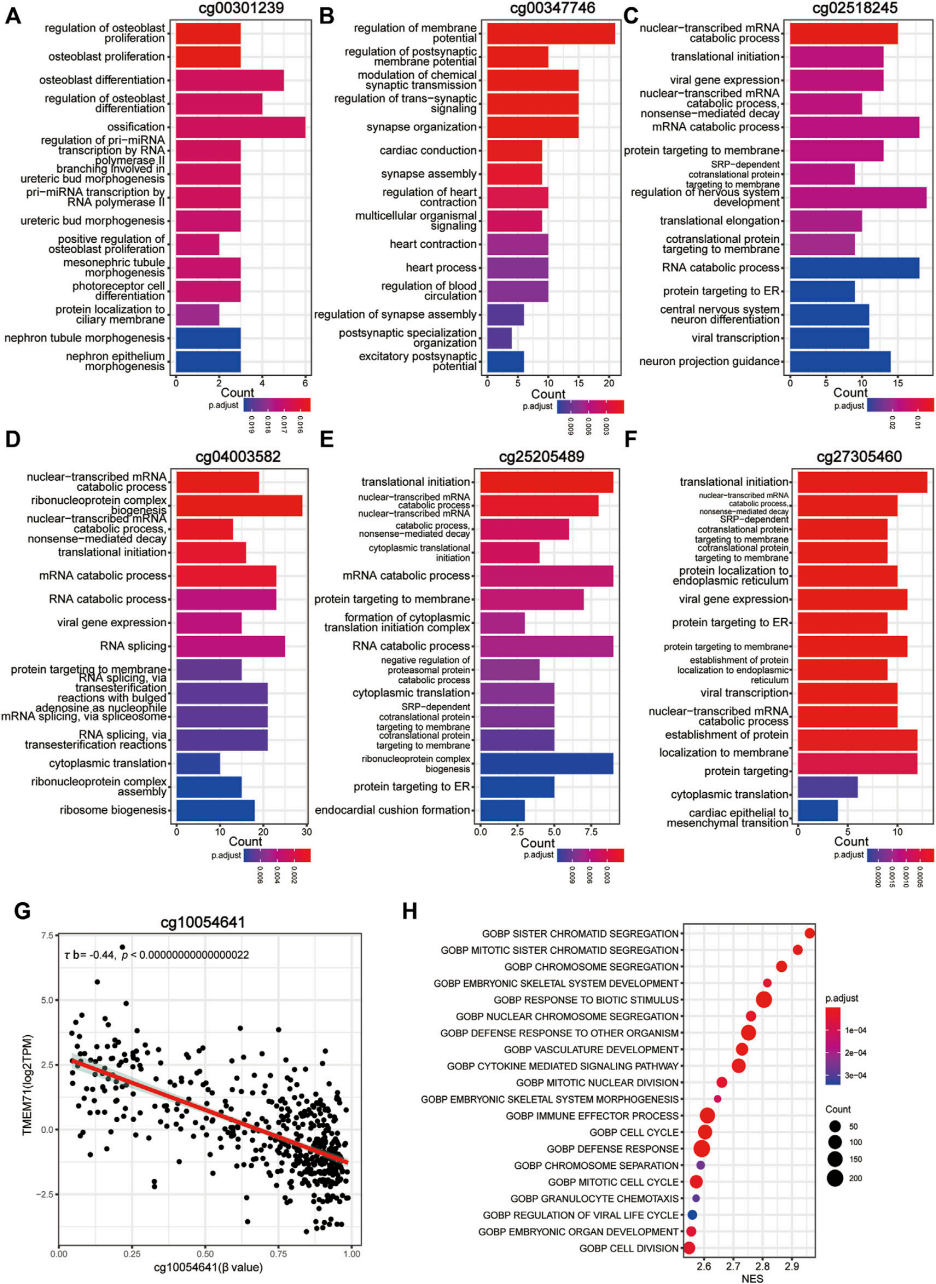
Functional characterization of the CpG signature. **(A**–**F)** Bar plot of GO annotation for genes with a strong correlation with non-promoter signature CpGs. **(G)** Correlation between methylation of cg10054641 and TMEM71 expression. **(H)** Dotplot of the GSEA results of the CpG signature.

**FIGURE 8:**
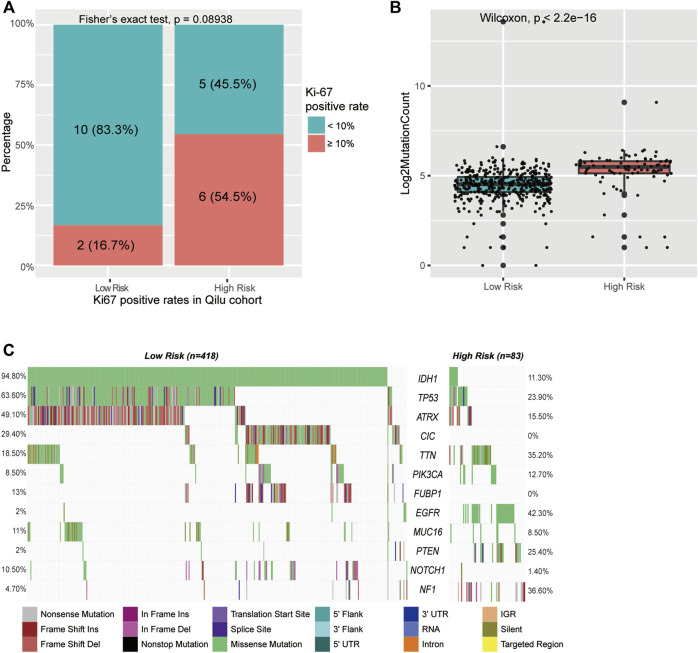
Mutations in CpG signature-stratified LGGs. **(A)** Barplot of Ki-67 level in different groups in the Qilu cohort. **(B)** Boxplot of mutation counts in the low-risk group and high-risk group. **(C)** Mutation landscape in the low-risk group and the high-risk group.

To better understand the biological basis of the metabolic CpG signature, we performed functional characterization of each signature CpG. Of the seven signature CpG sites, only cg10054641 was located in the promoter region of genes, and it was located in the TSS200 of TMEM71 (Table 2). To investigate whether cg10054641 regulates the expression of TMEM71, we calculated Kendall’s correlation between the methylation of cg10054641 and the expression of TMEM71. The results revealed a strong negative correlation (τb = −0.44, *p* < 0.05) between them, indicating that cg10054641 negatively regulates the expression of TMEM71 (Figure 7G). For the functional analysis of signature CpGs located in non-promoter regions, we screened genes with a strong correlation (|τb|>0.35, *p* < 0.05) with these CpGs and performed GO enrichment analysis. The results revealed that cg00347746 was correlated with genes that are mainly associated with normal cell functions such as synapse activities (Figure 7A), cg00301239 was correlated with genes that are involved in cell differentiation and proliferation and RNA processing (Figure 7B), and cg02518245, cg04003582, cg25205489, and cg27305460 were correlated with genes that are associated with RNA metabolism (Figures 7C–E). Next, we further conducted GSEA to investigate how the signature CpG genes function as a whole. First, using the “limma” R package, we conducted differential expression analysis on the high-risk group versus the low-risk group. Differences were considered significant if log2FC > 2 and *adjP* < 0.05. As a result, we identified 2028 DEGs between the two groups. After ranking these DEGs by their logFC value, we performed GSEA of these DEGs. The results showed that the high-risk group was significantly enriched in cell cycle- and immune-related processes (Figure 7H). Immunohistochemistry results of the Qilu cohort also revealed that tumours with higher risk scores were more likely to have higher Ki-67 positive rates (Fisher’s exact test, *p* = 0.08938), further confirming that the risk score was associated with proliferative activity. (Figure 8A).

**TABLE 2:** Annotation of the seven signature CpGs.

Probe	MAPINFO	Chromosome	UCSC_RefGene_Name	UCSC_RefGene_Group
cg00301239	103801487	8		
cg02518245	51573168	16		
cg04003582	92936507	5		
cg27305460	29969096	15		
cg25205489	24125993	13		
cg10054641	133773093	8	TMEM71; TMEM71	TSS200; TSS200
cg00347746	48970082	19		

Active cell mitosis is correlated with a high mutation burden and immune infiltration. To investigate the mutation characteristics of different risk groups, we performed an analysis of somatic mutation data from the TCGA cohort. A significant difference in mutation frequency between the high-risk group and the low-risk group was found (Figure 8B). The high-risk group, with active cell proliferation, was significantly correlated with higher mutation frequencies than the low-risk group. Next, the mutation patterns of the high-risk group and the low-risk group were also explored. Only mutations existing in more than 5% of all samples were analysed in this step. We found distinct mutation patterns between the high- and low-risk groups. IDH1 (11.3 vs. 94.8%, *p* < 0.001), ATRX (15.5 vs. 49.1%, *p* < 0.001), CIC (0 vs. 29.4%, *p* < 0.001), FUBP1 (0 vs. 13%, *p* < 0.001), and NOTCH1 (1.4 vs. 10.5%, *p* = 0.011) had significantly higher mutation rates in the low-risk group, whereas TTN (35.2 vs. 18.5%, *p* = 0.011), EGFR (42.3 vs. 2%, *p* < 0.001), PTEN (25.4 vs. 2%, *p* < 0.001), and NF1 (36.6 vs. 4.7%, *p* < 0.001) had significantly higher mutation rates in the high-risk group (Figure 8C and Supplementary Table S5).

### 3.7 The Metabolic CpG Signature Predicts Immune Infiltration and Potential Immunotherapy Response

**FIGURE 9:**
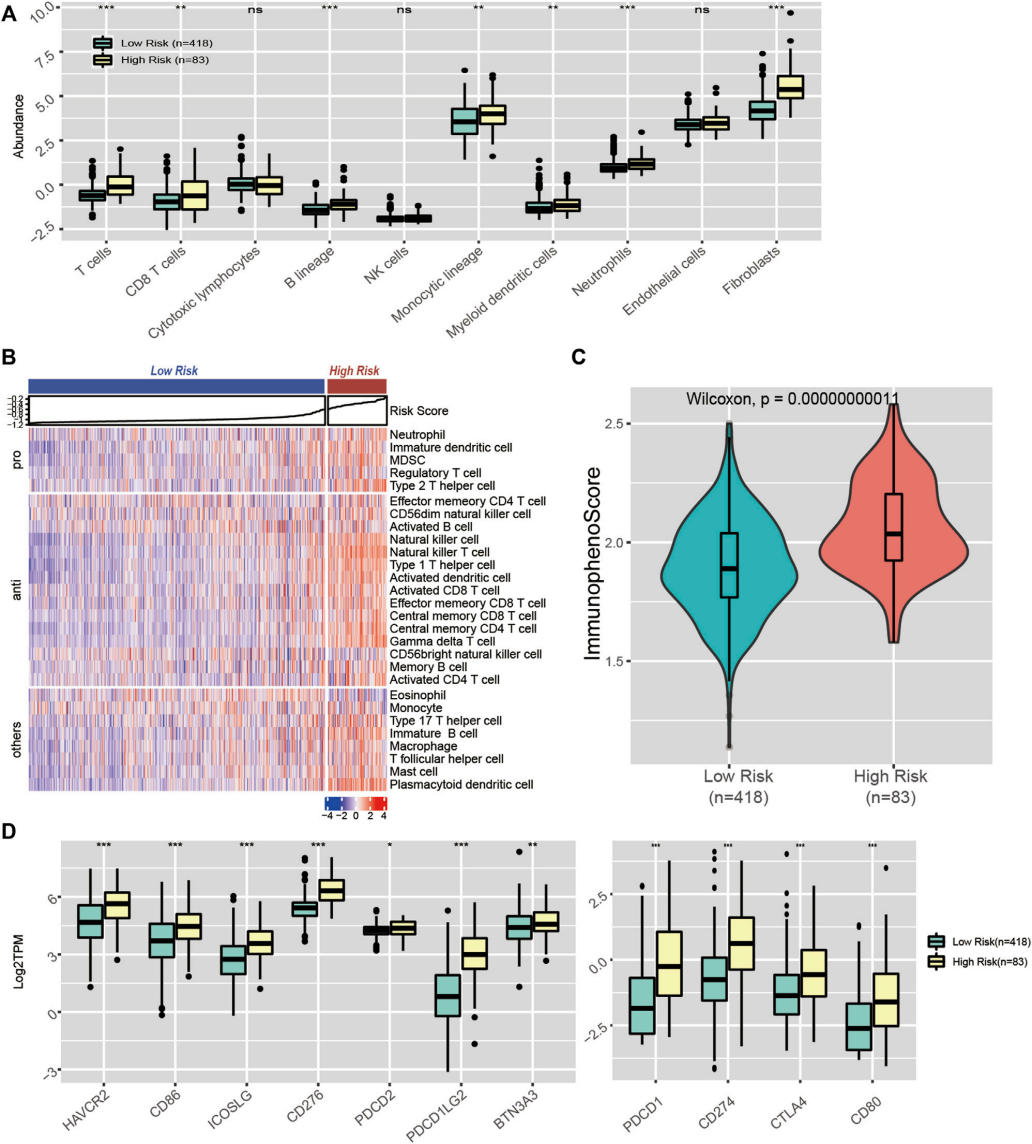
Immune landscape in CpG signature-stratified LGGs. **(A)** Boxplot of the abundance of 10 immune cell subsets estimated by MCP-counter. **(B)** Heatmap of the enrichment scores of 28 immune cell subsets generated by ssGSEA. **(C)** Violin plot showing the distribution of immunophenoscores in the low-risk and high-risk groups. **(D)** Expression levels of 11 immune checkpoint genes in the low-risk and high-risk groups.

We also evaluated the association between our signature and immune infiltration. First, we quantified 8 major immune subpopulations using MCP-counter and investigated their association with the risk score. We noticed that the risk score was accompanied by distinct immune infiltration. Compared with the low-risk group, the high-risk subgroup was infiltrated with significantly higher levels of antitumour cell subpopulations, such as T cells and CD8 T cells (Figure 9A). Next, we quantified 28 immune subpopulations with the ssGSEA algorithm. A similar result was observed. The high-risk group had significantly elevated levels of activated CD4 T cells, activated CD8 T cells, activated dendritic cells, central memory CD8 T cells, etc. (Figure 9B).

Immune checkpoints play important roles in the regulation of immune cell function. Therapy targeting ICIs has been considered the most promising antitumour treatment. Given that our signature was accompanied by distinct mutational loads and immune infiltration patterns, which are predictors of the response to ICI therapy, we assume that our signature could also be associated with the response to ICI therapy. Hence, we explored whether the distinct immune infiltration pattern between the high- and low-risk groups was accompanied by differences in ICI levels. As a result, we noticed a significant difference in immune checkpoint molecule expression between the two groups. Relative to the low-risk group, the high-risk group had significantly higher levels of PD-L1 (CD274), CTLA-4, and HAVCR2 (Figure 9D). Moreover, we quantified the potential immunotherapy response using the IPS algorithm and found that the IPS was significantly elevated in the high-risk group, which indicated that the high-risk group patients were more likely to respond to immunotherapy (Figure 9C). Overall, our signature could serve as a biomarker to predict patients’ response to ICI therapy.

## 4 Discussion

Due to the substantial heterogeneity of LGGs, even with identical histological diagnoses, patients usually have significant differences in prognosis. The conventional classification of gliomas solely based on histology can no longer satisfy patient stratification in the era of precision medicine. In recent years, many emerging molecular biomarkers have been identified. IDH mutation and chromosome 1p/19q codeletion were first incorporated into the 2016 WHO classification for the stratification of gliomas (Louis et al., 2016). Recent studies have highlighted the role of metabolic reprogramming in cancers (Faubert et al., 2020; He et al., 2020). Much effort has been devoted to exploring the metabolic heterogeneity of tumours from the perspective of transcriptomics and metabolomics. DNAm and metabolism have been proven to be closely interlinked (Lopes, 2020; Sun et al., 2021); however, no one has studied the value of the interplay between DNAm and metabolism in the stratification of gliomas. In the present study, we comprehensively analysed the correlation between metabolic pathways and DNAm. Based on metabolism-associated CpGs, we identified three distinct subgroups of LGGs and constructed a subgroup-related CpG signature. The molecular and clinical features of each subgroup/risk group were characterized. Our findings provide a new perspective for the stratification of LGGs and for understanding the mechanisms driving the malignancy of LGGs.

We first investigated the correlation between DNAm and 87 metabolic processes and acquired 708 metabolic process-CpG pairs consisting of 35 metabolic pathways and 391 CpGs. Based on these metabolism-associated CpGs, three distinct subgroups of LGGs were identified. Each subgroup had distinct clinical and molecular characteristics. Subgroup 1, characterized by high methylation of metabolism-associated CpGs, had a favourable outcome and low immune infiltration and was significantly correlated with IDH mutation, lipid metabolism and glutamine metabolism. Subgroup 3, characterized by low metabolism-associated CpG methylation, had the worst prognosis and high immune infiltration and was significantly enriched in cell cycle- and immune-associated processes, carbohydrate metabolic processes, and glycosylation processes. Subgroup 2, with moderate metabolism-associated CpG methylation, was correlated with embryonic development processes and had a moderate prognosis. Glycosylation, lipid metabolism, and carbohydrate metabolism are all considered important mechanisms promoting malignancy and novel therapeutic targets, suggesting that the subgrouping serves as a potential decision aid for the choice of treatment (Roth et al., 2020; Thomas et al., 2021). Considering that lipid metabolism is recognized as an important mechanism of promoting malignancy (Cheng et al., 2018), the positive correlation between lipid metabolism and better prognosis we revealed can be a little counterintuitive. Actually, this result is compatible with previous studies showing that phenotypes with upregulated lipid metabolism usually indicate a more favourable outcome, while phenotypes with upregulated carbohydrates indicate the opposite (Peng et al., 2018). A reasonable explanation could be that, unlike studies that focus on single variables, these analyses were performed based on real-world patient data and were affected by many other variables, the effect of lipid metabolism did not play a dominant role in this context. The recent cIMPACT-NOW update has recommended that for IDH wild-type gliomas, even if the histology suggests low grade, if accompanied by at least one of the three high-risk events, including chromosome 7gain/10loss, TERT promoter mutation, and EGFR amplification, they should be assigned to grade IV (Brat et al., 2018). Notably, in our results, more IDH wild-type LGGs with the above risk factors were assigned to subgroup 3, which indicated poor prognosis. This result suggested that metabolism-associated DNAm patterns could identify IDH wild-type high grade gliomas that were inappropriately assigned to lower grade according to previous criteria.

In glioma, genetic alterations, such as IDH mutation and EGFR amplification, are important contributors in shaping the tumour metabolic landscape. Mutated IDH catalyses the transformation of isocitrate into 2-HG, causing the accumulation of 2-HG and loss of α-ketoglutarate (α-KG), making the cell more dependent on glutamate-derived α-KG (Dang et al., 2009). BCAT1/2-catalysed conversion of branched amino acids and glutaminolysis are major sources of glutamate. However, in the context of IDH mutation, BCAT1/2 are inhibited by 2-HG, suggesting glutaminolysis as the major source of glutamate in IDH mutant LGGs (McBrayer et al., 2018; Wei et al., 2020). Consistently, subgroups 1 and 2 in our results were enriched with IDH mutation and had a higher level of D-glutamine and D-glutamate metabolism. IDH mutation has also been shown to impact lipid metabolism. For instance, although it inhibits *de novo* fatty acid synthesis at the substrate level by NADPH depletion, it also upregulates the levels of genes and proteins involved in fatty acid biosynthesis (Badur et al., 2018; Stuani et al., 2018; Dekker et al., 2020); it accelerates LDLR degradation to reduce exogenous cholesterol uptake, it also stimulates intracellular cholesterol biosynthesis (Stuani et al., 2018). In agreement with previous knowledge, our study revealed higher levels of fatty acid and cholesterol biosynthesis in subgroup 1 and subgroup 2. Moreover, IDH mutation could induce hypermethylation of the promoter regions of glycolysis genes, such as LDHA or CA9, thereby suppressing carbohydrate metabolism (Chesnelong et al., 2014). A shift into a G-CIMP-low phenotype or solely loss of DNA methylation at these specific loci can cause the acquisition of the Warburg phenotype (Ruiz-Rodado et al., 2020). EGFR amplification is a powerful driver of carbohydrate metabolism. It activates both AKT-dependent and AKT-independent pathways involving MYC and mTORC2 and ERK-dependent nuclear translocation of phosphorylated PKM2, thus upregulating glycolysis (Yang et al., 2012; Babic et al., 2013; Masui et al., 2013). Consistently, in our work, subgroup 3 mainly consisted of IDH mutant lesions with a G-CIMP-low phenotype and IDH wild-type lesions with EGFR amplification, and presented a higher level of carbohydrate metabolism than subgroup 1 and subgroup 2.

To make our results more convenient for practical application, based on the metabolism-associated CpGs that were differentially methylated across all subgroups, using LASSO Cox regression, we constructed a CpG risk signature that is quantifiable, applicable to individual patients and requires fewer features, which we termed the metabolic CpG signature. We assigned patients to a high-risk group and a low-risk group according to the signature. Survival analysis suggested that the metabolic CpG signature was capable of stratifying the prognosis of LGG patients in all three (TCGA/GEO/CGGA) cohorts. It was identified as an independent prognostic factor and has great prognostic power. Even when stratified by many major prognostic variables, our signature was still capable of further stratifying the prognosis of LGGs. To understand the underlying mechanisms of the signature, we performed some functional analyses. First, to understand how the signature functions as a whole, we performed GSEA analysis. The result indicated that the high-risk group was significantly enriched in cell cycle- and immune-related processes compared with the low-risk group. Further characterization of these signature CpGs was also performed. Of the seven signature CpG sites, only cg10054641 is a promoter CpG and regulates TMEM71 expression, which has been proven to be associated with malignancy and TMZ resistance in glioma (Wang et al., 2019). For nonpromoter signature CpGs, GO enrichment analysis was performed. The results indicated that cg00347746 is associated with normal cell functions, such as synapse activities, while cg00301239 is correlated with genes that are involved in cell differentiation, proliferation and RNA processing. cg02518245, cg04003582, cg25205489, and cg27305460 are mainly associated with RNA metabolism, which has been proven to be involved in the immune response (Nussbacher et al., 2019) and maintaining cellular metabolism (An and Duan, 2022; Peng et al., 2022).

Recently, ICI treatment has been considered the most promising antitumour treatment (Sanmamed and Chen, 2018). Nevertheless, not all individuals respond well to such treatment. Recent studies suggest that the degree of immune cell infiltration is critical for the response to ICI treatment (Herbst et al., 2014; Tumeh et al., 2014). Tumours with high immune cell infiltration tend to respond well to ICI therapy, whereas tumours with low immune cell infiltration tend to respond poorly (Herbst et al., 2014; Chen and Mellman, 2017). In our study, we revealed that the high-risk group was infiltrated with significantly higher degrees of antitumour immune cells (e.g., CD8 T cells) than the low-risk group, indicating that the high-risk group lesions belonged to an immune-inflamed phenotype. Major regulators of immune infiltration include TMB and cellular metabolism (Wang et al., 2022). TMB is positively correlated with neoantigens, which are recognized as key drivers of immune infiltration (Chalmers et al., 2017). In contrast, the IDH mutant-derived aberrant metabolite 2HG is recognized as a potent suppressor of the recruitment, activation and proliferation of antitumour T cells (Bunse et al., 2018). The difference in immune landscape between the two groups could be attributed to the high TMB in the high-risk group and the high IDH mutation rate in the low-risk group. In addition, the response to immunotherapy requires the presence of immune check points. The high-risk group also presented higher immune checkpoint expression, suggesting a good response to ICI therapy in the high-risk group. Finally, analyses using the IPS algorithm also drew consistent conclusions. In summary, the CpG signature could serve as a biomarker to predict the potential response to immunotherapy in LGGs.

Very interestingly, our results revealed that lipid metabolism is strongly correlated with the methylation of a significantly wider range of DNAm sites than other types of metabolism (Supplementary Figures S1A,B). Aside from the roles of being regulated, whether or how lipid metabolism participates in the regulation of tumour DNAm seem to have been unappreciated, whereas amino acid and carbohydrate metabolism have been proven to affect DNAm by regulating one-carbon metabolism or TETs (Xu et al., 2011; Rosenzweig et al., 2018). However, we noticed some clues that lipid metabolism might also regulate DNA methylation. One possible mechanism is that it might also regulate DNAm by lipid metabolism-related metabolites. For example, SAM is a major methyl group donor of the methylation process. The synthesis of phosphatidylcholine (PC) requires the methylation of phospholipid phosphatidylethanolamine (PE) and consumes SAM. This process is recognized as a major SAM consumer and serves as a methyl sink (Ye et al., 2017). PPARα, as a lipid homeostasis regulator, can be activated by certain lipid metabolites (Varga et al., 2011). It is also reported to affect DNAm by regulating the level of DNMT1(Luo et al., 2019). In addition, histone acetylation has also been reported to direct demethylase activity, and inhibition of histone deacetylase can cause replication-independent demethylation of DNA (Cervoni and Szyf, 2001; Ou et al., 2007). It is well established that histone acetylation can be regulated by lipid metabolism in many ways. The lipid-derived metabolite acetyl-CoA serves as a major source of carbon for histone acetylation (McDonnell et al., 2016). An end-product of fatty acid metabolism, β-hydroxybutyrate (β-OHB), is recognized as a histone deacetylase (HDAC) inhibitor (Shimazu et al., 2013). Another possible mechanism is that fatty acid synthase (FASN) might directly participate in DNAm. This lipid metabolic enzyme has been reported to have a methyltransferase domain and be able to localize to the nucleus (Maier et al., 2006; Madigan et al., 2014). However, whether this domain is functional remains unknown. Anyway, the strong covariation between lipid metabolism and DNAm is interesting and worth exploring. Future work can further delineate the molecular details of how lipid metabolism is linked to the regulation of DNAm. This might provide a new perspective on how metabolism drives malignancy.

In our work, we explored the relationship between metabolic reprogramming and DNAm from the perspective of the transcriptome and methylome and performed our analyses based on this relationship. However, the limitation of our study is that transcriptional changes do not fully reflect all metabolic changes. Other variables, such as protein modification status, also play important role. Therefore, to investigate the crosstalk between DNAm and metabolism more comprehensively, it is better to further incorporate metabolomic data and proteomic data for analysis.

In summary, our analysis identified three metabolism-associated DNAm subgroups in LGGs and constructed a CpG signature to stratify prognosis and to predict the response to ICI treatment. Our findings provide new perspectives for the stratification of LGGs and highlight the role of DNAm in metabolism-associated tumor heterogeneity.

## Data Availability

The datasets presented in this study can be found in online repositories. The names of the repository/repositories and accession number(s) can be found in the article/Supplementary Material.
