# Supplementary figures and images for: Metabolism-Associated DNA Methylation Signature Stratifies Lower-Grade Glioma Patients and Predicts Response to Immunotherapy
Source: Front Cell Dev Biol. 2022 Jun 15;10:902298. doi: 10.3389/fcell.2022.902298 (PMC9240391; doi:10.3389/fcell.2022.902298)

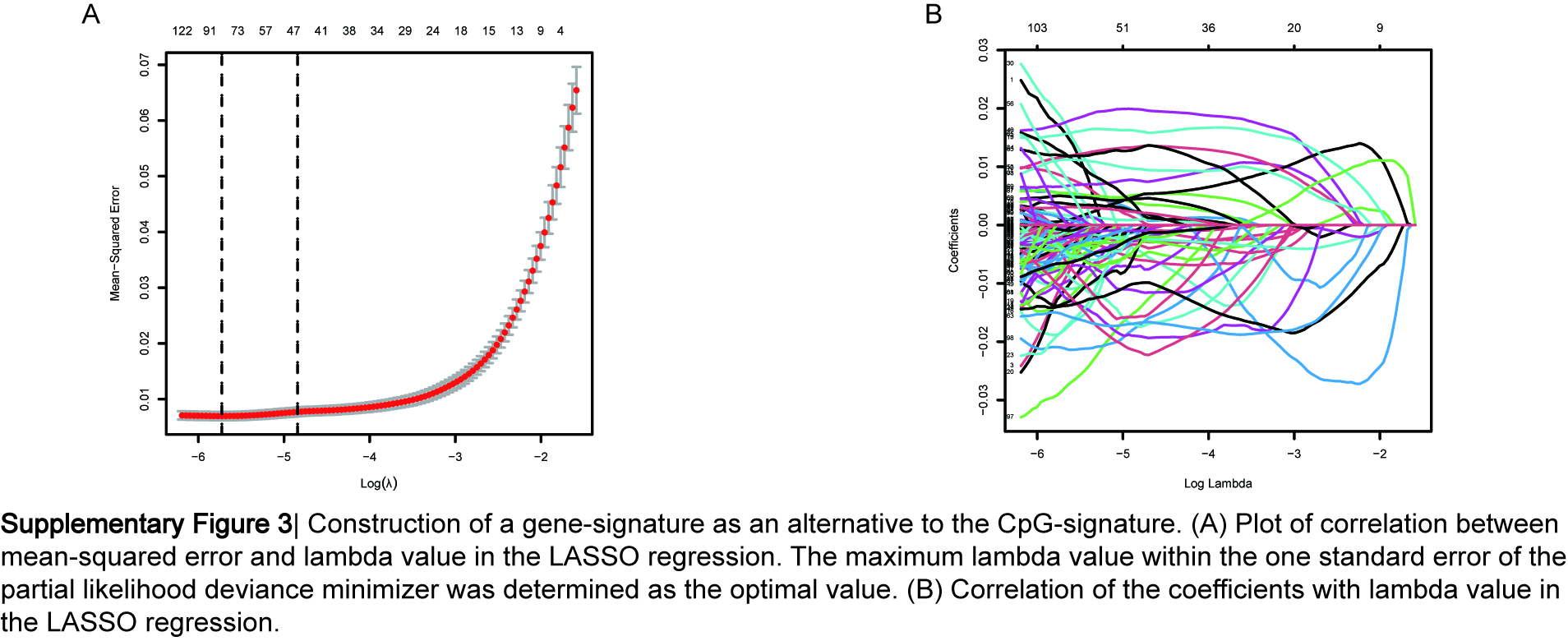

Supplement: Supplementary file 3 [file Image3.TIF]

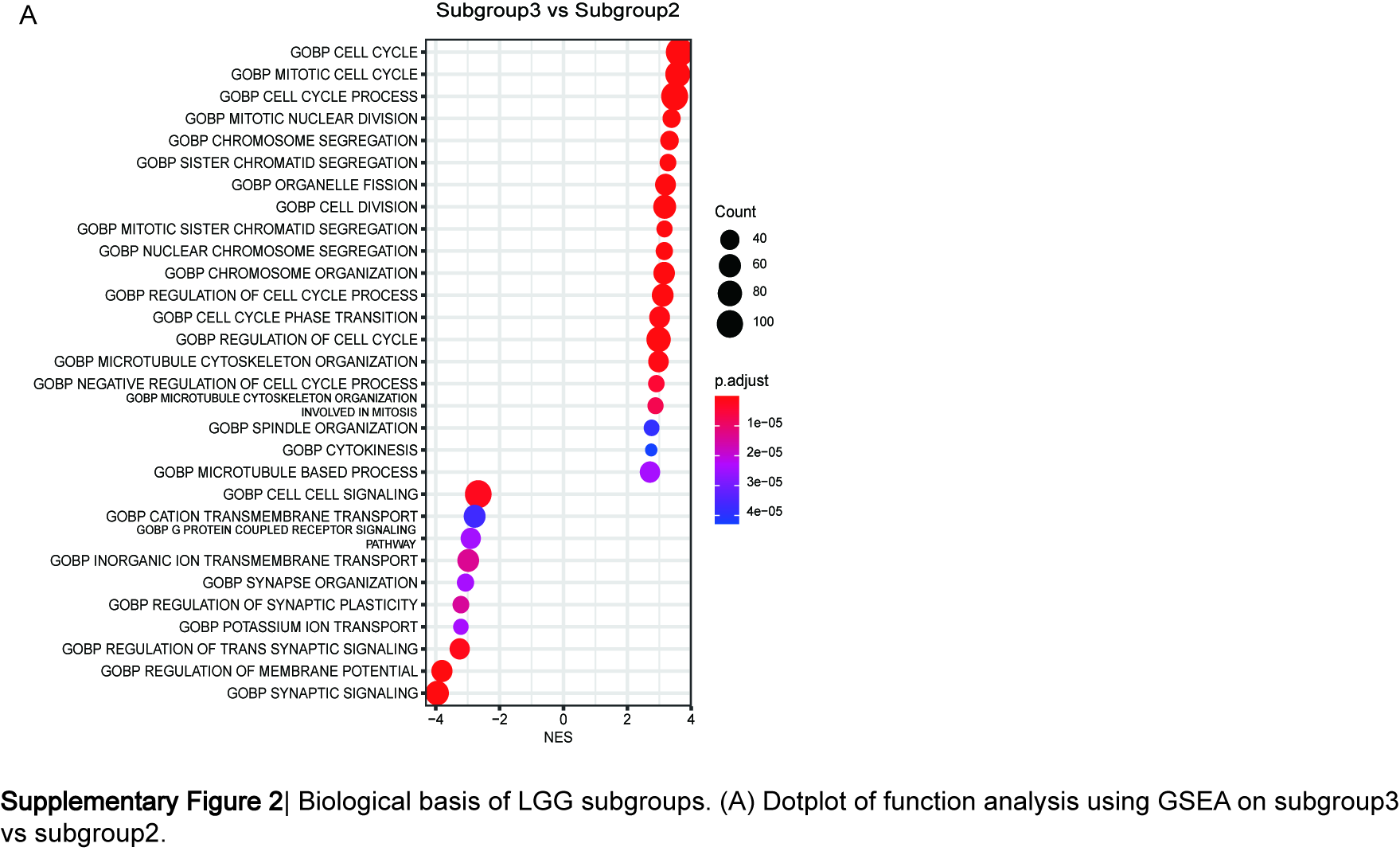

Supplement: Supplementary file 4 [file Image2.TIF]

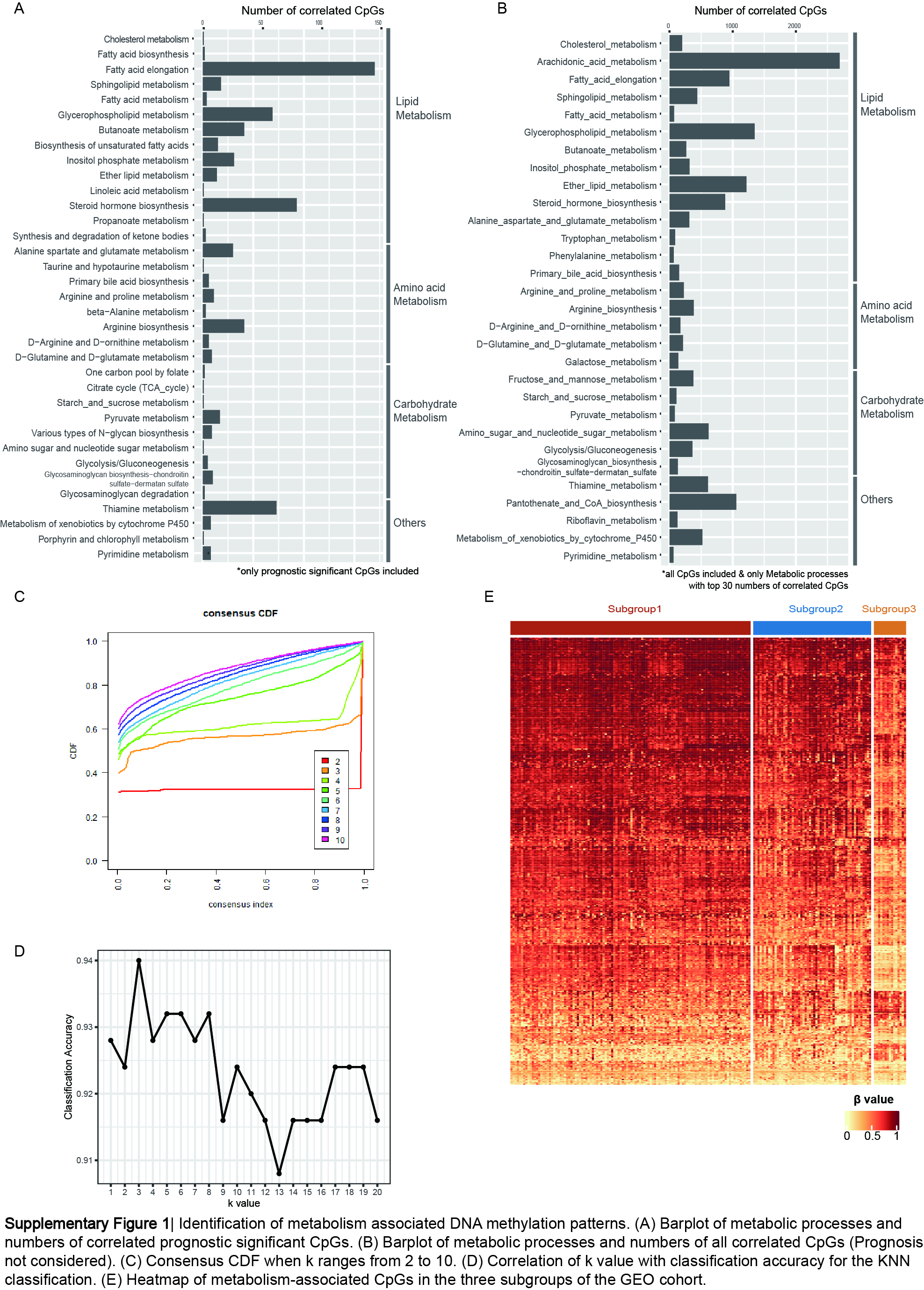

Supplement: Supplementary file 5 [file Image1.TIF]
